# Supplementary material for: New approach to improve power consumption associated with blockchain in WSNs
Source: PLoS One. 2023 May 23;18(5):e0285924. doi: 10.1371/journal.pone.0285924 (PMC10205006; doi:10.1371/journal.pone.0285924)
Supplement: Attachment — Submitted filename: Response to Reviewers.docx [file pone.0285924.s001.docx]

March 9, 2023

Dear Editor:

Please find enclosed several attached files corresponding to the amended version of our research paper entitled:

*“New approach to improve power consumption associated with blockchain in WSNs”*

It has been revised following the recommendations of the reviewers, all major changes being highlighted in red.

In the following paragraphs we attend to the suggestions that you made regarding formal aspects of the article. We want to express our gratitude for your comments, which will significantly increase the quality of our article.

Yours sincerely,

Maytham S. Jabor

Institute ITACA of Information and Communication Technologies

Universitat Politècnica de València

46071 Valencia (SPAIN)

[maytham.jebory@gmail.com](mailto:maytham.jebory@gmail.com)

Academic Editor

Thanks for your useful comments. Following your instructions, we have carried out the actions mentioned below:

***1. Please ensure that your manuscript meets PLOS ONE's style requirements, including those for file naming. The PLOS ONE style templates can be found at*** [***https://journals.plos.org/plosone/s/file?id=wjVg/PLOSOne_formatting_sample_main_body.pdf***](https://journals.plos.org/plosone/s/file?id=wjVg/PLOSOne_formatting_sample_main_body.pdf) ***and*** [***https://journals.plos.org/plosone/s/file?id=ba62/PLOSOne_formatting_sample_title_authors_affiliations.pdf***](https://journals.plos.org/plosone/s/file?id=ba62/PLOSOne_formatting_sample_title_authors_affiliations.pdf)

The manuscript has been upgraded to meet the journal format.

***2. Please note that PLOS ONE has specific guidelines on code sharing for submissions in which author-generated code underpins the findings in the manuscript. In these cases, all author-generated code must be made available without restrictions upon publication of the work. Please review our guidelines at*** [***https://journals.plos.org/plosone/s/materials-and-software-sharing#loc-sharing-code***](https://journals.plos.org/plosone/s/materials-and-software-sharing#loc-sharing-code) ***and ensure that your code is shared in a way that follows best practice and facilitates reproducibility and reuse.***

The data and code have been uploaded to a public repository that can be found at <https://github.com/Aqeel9090/-Improve-Wireless-Sensor-Network-with-Blockchain->

***3. PLOS requires an ORCID iD for the corresponding author in Editorial Manager on papers submitted after December 6th, 2016. Please ensure that you have an ORCID iD and that it is validated in Editorial Manager. To do this, go to ‘Update my Information’ (in the upper left-hand corner of the main menu), and click on the Fetch/Validate link next to the ORCID field. This will take you to the ORCID site and allow you to create a new iD or authenticate a pre-existing iD in Editorial Manager. Please see the following video for instructions on linking an ORCID iD to your Editorial Manager account:*** [***https://www.youtube.com/watch?v=_xcclfuvtxQ***](https://www.youtube.com/watch?v=_xcclfuvtxQ)

The ORCID of the corresponding author is 0000-0002-4518-0290.

***4. Please amend your authorship list in your manuscript file to include author Maytham Jabor.***

The author list has been corrected.

***5. We note that Figures 2, 9 and 10 in your submission contain copyrighted images. All PLOS content is published under the Creative Commons Attribution License (CC BY 4.0), which means that the manuscript, images, and Supporting Information files will be freely available online, and any third party is permitted to access, download, copy, distribute, and use these materials in any way, even commercially, with proper attribution. For more information, see our copyright guidelines:*** [***http://journals.plos.org/plosone/s/licenses-and-copyright***](http://journals.plos.org/plosone/s/licenses-and-copyright)***.***

I am afraid that this is not correct. Picture 2 has been done by the authors, despite of the fact that it is very similar to many other pictures that describe cluster-based architectures. It has been slightly modified to make different from other pictures . In addition, we have added a reference to the figure 2. As proof, the original PowerPoint file is also included in this submission.

Regarding pictures 9 and 10, the figures have been removed.

***6. Please include captions for your Supporting Information files at the end of your manuscript, and update any in-text citations to match accordingly. Please see our Supporting Information guidelines for more information:*** [***http://journals.plos.org/plosone/s/supporting-information***](http://journals.plos.org/plosone/s/supporting-information)***.***

The figure captions have been included at the end of paper, as requested.

Additionally, the statistical analysis has been enhanced in the paper to provide more information about the compression ratio. In this sense, the use of SPSS tool and a new figure 8 and table 2 are provided, being also commented in the text.

The use of English has also been improved.

Thanks for those useful comments. Please, do not hesitate to ask any additional questions that may arise.

Please find below the letters that address the issues from the reviewers

Kind regards

Maytham S. Jabor

Reviewer #1:

Dear reviewer:

First of all, thanks for evaluating this article. Your comments have been very useful to improve our paper. The revised manuscript includes your suggestions. Our amendments are highlighted to ease your work. Your questions are addressed below:

Note: Please, put in your mind that the order of the references sequence has been modified to be an arranged and easier manner for the reader.

***1)In my opinion, the author should better discuss their result to stress what are the advantages of using blockchain. For example, they could insert some results about the network's resilience to churn nodes.***

Thanks for your comment. New paragraphs have been included in the introduction to point the advantages that Blockchain may provide to WSN. Many of the references in the State of Art section also address these advantages. Nevertheless, in this step, we focus on the energy consumption of the wireless sensor network. Our Future work section also foresees the evaluation of network performance under the threat of many types of attacks. It is expected to perform series of experiments where the network behavior will be monitored while under these attack profiles.

***2)In the Introduction, the authors said that speed is one of the advantages of the blockchain. Could they explain why? Usually, the transaction speed is one of the blockchain limitations.***

Thanks for your comment. Some authors have compared the performance of Blockchain technologies in WSN in front of centrally controlled ledger. According with this, Blockchain removes any third-party intervention between transactions and removes mistake and make system efficient and faster.

In this sense, a new reference [10] has been included, and discussed in the text

*[10] Golosova and A. Romanovs, "The Advantages and Disadvantages of the Blockchain Technology," 2018 IEEE 6th Workshop on Advances in Information, Electronic and Electrical Engineering (AIEEE), Vilnius, Lithuania, 2018, pp. 1-6, doi: 10.1109/AIEEE.2018.8592253.

***3)In Section Proposed System, the authors talk about the POS consensus algorithm. Is it proof of stake? It needs to be clarified how they use it.***

Thanks for your comment. POS (Proof of Stake) is a well-known consensus algorithm. The text has been rewritten to clarify this point and describe the basic performance of this algorithm when creating and spreading a new block.

***4) The author should better comment on Figures 15 and 16 to stress the advantages of their model compared to the others*.**

Thanks for your comment. Its now (Figures 13 and 14) illustrate the network lifetime, comparing hardware and software implementations with the LEACH protocol case without Blockchain (red color line). Obviously, the use of Blockchain increases the energy consumption, thus reducing the network lifetime. Traditional software Blockchain is represented in brown color. Our proposal (both hardware and software approaches) is closer to maximum lifetime than software Blockchain. The text has been rewritten to enlighten these differences.

***5)The Authors should share their data and codes to allow everyone to reproduce their results.***

Thanks for your comment. The data has been uploaded to a public repository that can be found at <https://github.com/Aqeel9090/-Improve-Wireless-Sensor-Network-with-Blockchain->

***6) [30], [31], [35] are websites and are better as footnotes.***

***- The quality of Figures 3, 6, 7, 8, and 9 has to be increased.***

***7) The arrow size of Figure 2 should be increased.***

***8) Figure 5 is cited before Figure 4; they should be inverted.***

Thanks for your comments. They have been applied to the text, and the references have been corrected. About The figure 9 has been replaced by a table..

***9) Section Circuit design and implementation should start with an introduction and not directly with a subsection.***

Thanks for your comment. An appropriate introduction paragraph has been included in the text.

Finally, we would like to express our appreciation for the useful feedback you have provided us. It has been essential to increase the quality of our work.

Kind regards

Maytham S. Jabor

Reviewer #2:

Dear reviewer:

First of all, thanks for evaluating this article. Your comments have been very useful to improve our paper. The revised manuscript includes your suggestions. Our amendments are highlighted to ease your work. Your questions are addressed below:

Note: Please, put in your mind that the order of the references sequence has been modified to be an arranged and easier manner for the reader.

1) ***a clear definition of security property should be added It should be analyzed whether the level of security is increased, which security properties are provided, and the overall benefits from a security point of view.***

Thanks for your comment. The text has been modified to address the security properties and the general benefits provided by Blockchain from the security point of view. The increase in the security level has enlightened in the text, including a new reference [12].

***2) citations of relevant concepts are missing (i.e. the form “Most researchers” must be followed by the associated references, “Leach protocol” in the “Proposed system” section, “Verilog” in the “Circuit design” section) or a not correctly placed (i.e. the 8th source)***

Thanks for your comment. Multiple references have been included throughout the text that support the statements of the text. Also LEACH protocol [44,45] and wrong references have been corrected. We apologize for these mistakes.

***3) several sentences are too long and this affects the understanding of the concepts/ideas***

***4) certain concepts that are repeated***

***5) the use of “This way” should be dramatically reduced***

***6) the use of brackets should be limited. They should not contain important concepts (i.e. “the hash of the current block” in the “Introduction” section).***

***7) the English should be improved (there are several grammatical and punctuation errors that should be avoided)***

***8) the style of the article is not coherent (so bulleted lists, approaches name, and acronyms should be written in the same way)***

Thanks for your comments, and we apologize for these mistakes. The text has been deeply revised, correcting the style, use of English, format issues, avoiding fillers such as “this way” when possible, and improving both punctuation and grammatical aspects.

***9) the advantages of a concept (such as blockchain technology, or chaos theory) should be inserted after its definition. The exchange will increase the clarity of the paper and its basic notion.***

Thanks for your comments. The text has been rearranged to approximate the definition of a concept and its advantages. Proper references have been also added.

***10)basic concepts on BC and chaotic theory could be introduced in a suitable “Background” section.***

Thanks for your comments. The main theory of Compressive Sensing using Chaos is cited in references [22-29]. A brief introduction of main concepts was explained in the Introduction Section. A deeper revision of these theory at this point may confuse the reader.

***Below we provide a list of the issue for each section that should be addressed:***

**- In “Abstract”:**

- ***it should be interesting to have a clear motivation about how this approach added confidentiality and integrity. (It could be inserted in one of the sections of the paper but at the current stage is missing.).***

Thanks for your comment. New paragraphs have been included in the introduction to point the advantages that Blockchain may provide to WSN. Many of the references in the State of Art section also address these advantages, proposing different confidentiality and integrity mechanisms based on Blockchain. Nevertheless, in this step, we focus on the energy consumption of the wireless sensor network.

**- In “Introduction:**

- ***some ideas are unclear (such as the adoption of BC in WSNs and why it is a successful strategy in the use of chaos on hardware).***

Thanks for this comment. The writing of the introduction has been deeply modified to clarify these concepts and some others that we were not able to explain properly. Also, references [30], [31] and [34] has been added to provide more detailed information about each of the ideas.

- ***Nakamoto’s paper should be cited when introducing the notion of BC certain definitions are missing. In particular, we suggest introducing the blockchain as a distributed ledger because this concept is used in the BC advantages and in the “Proposed system” section.***

We consider this comment very useful. It has been added as reference [6]. Also the concept of distributed ledger has been also rewritten and a new reference [8] has been included

**- In “State of the art”:**

- ***the illustrated existing approaches must achieve energy consumption (as the introductory sentence explains) but the 25th source is inserted even if it does not fulfill this goal. Its inclusion should be motivated.***

Thanks for this comment. This reference is now [38]. In this paper an energy-efficient clustering approach based on blockchain technology for WSN network is presented. Its approach is similar to [41], although Blockchain consumption is not the center of the paper.

- ***all the sources should be classified according to some criteria that are a priori defined (i.e. including implemented methodology/ies, security properties provided, innovation/s) and it could be useful to insert a table that summarizes them****.*
- ***all the existing approaches should be compared with the researchers' one as it is done for the 28th source. If the 28th source is more relevant than the others should be motivated.***

Thanks for this comment. A new table 1 has been included to classify the different approaches, being also commented in the text.

Also, these approaches have been compared with our proposal. The appropriate paragraphs have been included in the Conclusion section.

**- In “Proposed System”:**

- ***the use of the figures is not effective (such as Fig.5 is cited in advance compared to its position)***

Thanks for this comment. The citation has been removed to preserve the logical sequence of the idea.

- ***some sentences could be simplified (such as “Nevertheless, …., encrypted”)***

Thanks for this comment. Many sentences, such as the described one, have been rewritten.

- ***the layout associated with the data flowing into the network should be improved. Fig.3 should be inserted before the overall description of the mechanism to better understand it.***

Thanks for this comment. The figures have been improved to increase their readability. Also, the order has changed into a more logical sequence.

**- In “Circuit design and implementation”:**

- ***the introductory sentence that illustrates the purpose of the section is missing***

Thanks for this comment. We agree with you. An introduction to this section has been added.

- ***the overall structure should be simplified because there are a lot of repetitions (such as the definition of the parameters should be listed once). Some basic ideas are already expressed in the “Introduction” section so are redundant.***

Thanks for this comment. The article has been thoroughly updated, take special care about the repetition of concepts. They have been removed.

- ***the Algorithm definition and analysis are not clear (such as the parameters could be better visualized using a bulleted list)***

Thanks for this comment. The description of the parameters has been improved into a bulleted list.

- ***there is a line spacing issue after Algorithm 3 and the explanation of this Algorithm could be improved.***

Thanks for this comment. The line spacing has been corrected. Also, we have improved the explanation of the algorithm's operation and its steps.

- ***the interpolation part should be rephrased and clarified when and why it is required to be used (the use of an example, 37th source, is not enough)***

Thanks for this comment. A brief explanation of the interpolation process has been added to the text. Also, new references [50], [51] and [52] may provide more examples.

**- In “Experimentation and Results”:**

- ***the acronym CS is incorrectly used. It stands for compressed sensing, not for signal compression. The use of the same acronym is misleading.***

Thanks for this comment. The indicated acronym CS has been removed in this context to avoid misleading.

- ***Fig. 9 and Fig. 10 could be replaced with tables showing the outcomes of the FPGA implementation to have clear evidence of the values.***

Thanks for this comment. The figures have been replaced by a table. A new table 3 has been included to enlighten the values.

- ***the analysis of how the microcontroller works at higher frequency should be added in the “Future work” section***

Thanks for this comment. It has been included in the Future Work section.

- ***Figures should be deployed and analyzed. In particular, Fig. 15 and Fig.16 show three different approaches, their results should be examined and the benefits (or the drawbacks) should be illustrated.***

Thanks for this comment. A discussion about figures 15 and 16 has been added. On the other hand, we consider interesting the comparison of network lifetime in the four supposals: without Blockchain (only Leach – red line), traditional software Blockchain (brown line) and hardware and software CS blockchain (blue lines).

**- In “Conclusions and future work”:**

- ***the definition of which type of attack will be analyzed as future work should be added.***

Thanks for this comment. We have added a new paragraph regarding this issue.

Finally, we would like to express our appreciation for the valuable feedback you have provided us. They have been very helpful to increase the quality of our work.

Kind regards

Maytham S. Jabor
